# The Impact of Surgery-Related Emotional Distress on Long-Term Outcome After Colorectal Surgery: An Observational Follow-Up

**DOI:** 10.3390/jcm13216406

**Published:** 2024-10-25

**Authors:** Ann-Kathrin Lederer, Ines Manteufel, Agnes Knott, Alexander Müller, Lampros Kousoulas, Paul G. Werthmann, Alexandra C. Klein, Roman Huber

**Affiliations:** 1 Center for Complementary Medicine, Department of Medicine II, Medical Center—University of Freiburg, Faculty of Medicine, University of Freiburg, 79106 Freiburg, Germany; 2 Department of General, Visceral and Transplant Surgery, University Medical Center of the Johannes Gutenberg University, 55131 Mainz, Germany; 3 Center of Surgery, Department of General and Visceral Surgery, Medical Center—University of Freiburg, Faculty of Medicine, University of Freiburg, 79106 Freiburg, Germany; 4 Institute for Applied Epistemology and Medical Methodology at the University of Witten/Herdecke, 79111 Freiburg, Germany; 5 Research Group Integrative Medicine, Department of General and Visceral Surgery, University Hospital Ulm, 89081 Ulm, Germany

**Keywords:** emotions, surveys and questionnaires, psychological distress, treatment outcome, colorectal surgery, postoperative complication, MDMQ

## Abstract

**Background**: Preoperative emotional distress has been linked to adverse health outcomes, diminished quality of life, increased symptom burden after surgery, and poorer postoperative outcomes. Therefore, this study aimed to assess the impact of perioperative emotional distress on the long-term outcomes of patients undergoing colorectal surgery. **Methods**: We conducted a follow-up study of a previously conducted observational study of colorectal surgery patients who underwent surgery at the University Medical Centre Freiburg, Germany, between April 2018 and February 2020. Initially, all the patients completed a multidimensional mood questionnaire (MDMQ) preoperatively and on postoperative days 3, 6, and 9. From June to November 2021, these patients were contacted again to capture the results of MDMQ at least one year after surgery and to assess the rate of late complications, quality of life, dietary changes, and overall health. **Results**: Of the original 80 patients, 51 took part in the follow-up study (55% female, on average 59 years old), on average 30 months after the operation. The average mood results of the surgical patients returned to those of the normal population. Most patients (80%) did not feel that their quality of life was affected by the surgery. Satisfaction with the surgical outcome averaged 8.5 out of 10, while current health was rated at 6.8 out of 10. Emotional distress levels varied over time, with mood and arousal improving significantly postoperatively. Late complications were reported by 28% of the patients, but there were no significant differences in the MDMQ scores, health status, or satisfaction between those with and without complications. **Conclusions**: The results indicate that there is no association between the emotional strain measured by the MDMQ and the occurrence of late postoperative complications.

## 1. Introduction

For most patients, the need for surgery is an existential borderline experience, as surgical interventions are associated with anxiety and the fear of dying [1]. Every operation is a massive physical and psychological distress for the human body, which have little in common with the normal physiological function of the human body [2,3]. Recent research suggests that preoperative distressing feelings can have a negative impact on the health status, the quality of life, and the symptom burden after surgery [4,5,6]. Psychological interventions before cancer operations have been reported to influence the psychological outcome and quality of life after surgery [7,8]. Around a quarter to a fifth of surgical patients have emotional stress scores that exceed the normal level [9,10]. These patients are at high risk for remaining distress after a surgical procedure and for the development of persistent psychiatric disorders [11]. In addition to these psychological effects, emotional stress could also have an influence on the functional outcome after surgery. It is known that stressful events can have a severe and potentially life-threatening impact on the body, such as the broken heart syndrome [12]. Furthermore, there is an ongoing debate about whether distress affects the outcome and survival of cancer patients [13,14,15,16,17]. In conventional medicine, the body is commonly separated from the mind, although a large variety of research works show that mind–body-based approaches can help alleviate physical symptoms such as fatigue, pain, and nausea [18,19,20]. In a systematic review, Paulsen et al. describe that some studies show evidence that the functional outcome after total knee replacement could be worse if the patients were emotionally stressed before the operation [21]. Maratos et al. reported that psychological distress does not comprise the outcome after spinal surgery, whereas newer publications indicate an effect of psychological comorbidities on the outcome [22,23]. Amaral et al. reported that emotional distress leads to a poorer outcome after spine operations, implying a lower quality of life and more severe physical disability [24]. Preoperative depression has been associated with poorer outcomes for patients undergoing neurosurgery [25,26]. Multimodal prehabilitation concepts, including mental preparation, can improve the outcome of patients undergoing major abdominal surgery [27,28]. Foster et al. stated in 2016 that preoperative self-efficacy and the level of depression can predict the outcome of colorectal cancer patients in terms of the quality of life, personal well-being, and health status [29]. Patients who have to undergo colorectal surgery are in a particularly stressful situation, as there is the risk of postoperative fecal incontinence and an artificial anus could be created as a consequence of the operation. Colorectal surgical procedures and the potential creation of an ostomy impact the body image of patients and are accompanied by feelings of shame and anxiety [30,31]. It is, therefore, important to identify the emotional strains of patients who undergo colorectal surgery. In a previous study, we evaluated the effects of emotional distress on the short-term outcome after colorectal surgery using a standardized multidimensional mood questionnaire [32]. Patients with and without short-term postoperative complications differed not significantly regarding their perioperative emotional strain, but we were able to map the perioperative emotional burden of patients. With this study, we have pursued the goal of obtaining the long-term outcome of colorectal surgery patients to investigate whether emotional distress had an influence on the long-term outcome after colorectal surgical procedures.

## 2. Materials and Methods

The current report is a follow-up study to a previously reported study that investigated the relationship between perioperative psychological distress and the short-term outcome after surgery [27]. The aim of the follow-up study was to investigate the influence of preoperative emotional distress on the long-term outcome after colorectal surgery. As previously reported, the initial study was conducted between April 2018 and February 2020 as a monocentric, paper-based observational study among inpatients undergoing elective colorectal surgery in the Department of General and Visceral Surgery at the Freiburg University Medical Center [27]. In brief, all the included patients completed a well-validated multidimensional mood questionnaire (MDMQ) preoperatively and on the 3rd, 6th, and 9th postoperative days [28,29,30,31,32]. The MDMQ was selected after consultation with the Department of Psychosomatic Medicine and Psychotherapy, as it is well suited for postoperative use due to its brevity. We assumed that the brevity and simplicity of the questionnaire would improve patient compliance. In Germany, the MDMQ is a widely used and well-validated questionnaire designed to assess a person’s emotional situation [33,34,35,36,37]. The questionnaire is only available in German. The questionnaire covers three oppositional dimensions (mood (bad vs. good), wakefulness (tired vs. awake), and arousal (nervous vs. calm)) by using 24 items, each with a five-point rating scale. The results range between 1 (feeling of maximum bad mood, tiredness, or nervousness) and 40 points (feeling of maximum good mood, readiness, or calmness) for each dimension. Examples of the items that can be captured by the MDMQ are happiness, well-being, calmness, relaxation, and serenity. The discriminative power of the individual items when healthy subjects were surveyed ranged from a minimum of 0.57 to a maximum of 0.88 [37]. Since the MDMQ is available for purchase, it is not possible to show the MDMQ in this manuscript.

All adult and German-speaking patients who underwent elective colorectal surgery (left hemicolectomy, right hemicolectomy, sigmoid resection, deep anterior rectal resection, and the restoration of continuity) without the primary intended creation of ostomy were eligible for inclusion. The patients who received an ostomy or underwent emergency surgery and patients with acute psychiatric illnesses were not included due to the possible influence on the MDMQ result.

**Figure 1:**
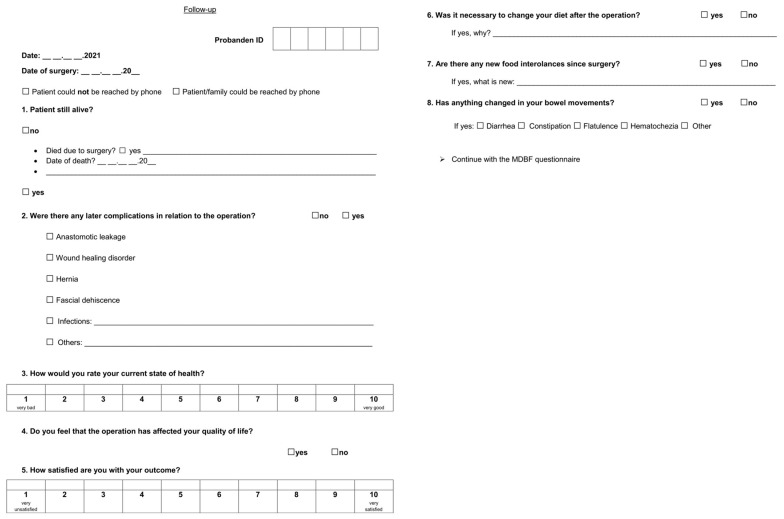
Follow-up interview strategy.

Although written consent was available for all the patients from the previous study, the patients were asked again by telephone whether they agreed to participate in the follow-up examination. All the patients initially analyzed (n = 80) were contacted again by telephone between June and November 2021 to record the outcome at least one year after surgery. For this purpose, we developed an interview strategy based on the recommendations of Hing et al., in which we asked about late complications such as healing disorders or hernias, and about quality of life, diet, and general health (see Figure 1) [38].

As there is no clear definition of a “late complication”, we recorded pathological conditions that could be related to the operation. We defined a late complication as what the patients themselves considered to be a stressful and debilitating consequence of the surgery (such as changes in bowel habits) or what led to further intervention (such as an incisional hernia). We also queried the MDMQ again at follow-up.

For each patient who could not be reached initially, there were several attempts to reach them by telephone over the following weeks. In addition to the personal interviews, the patients’ electronic patient records were examined with regard to the subsequent postoperative course. To find out whether patients had died, death notices in local newspapers were also taken into account.

The initial study and the follow-up study were approved by the local ethics committee (EK-FR: 535/17). The initial study was registered with the German Clinical Trials Registry (DRKS00014059). Both studies were conducted according to the principles of the Declaration of Helsinki and the ICH guidelines for good clinical practice (GCP).

### Statistical Plan

This was an exploratory follow-up study that was intended to reach as many patients as possible from the initial study. For the initial study, a sample size of 72 patients overall was calculated to detect a statistical difference of *p* < 0.05 between the MDMQ results of patients with and without postoperative complications with a statistical power of 80% and the medium effect size of one standard deviation [27]. The original primary aim was to determine the relationship between the patients’ mood as measured with the MDMQ and the rate of postoperative complications according to the Clavien–Dindo classification [39]. The primary target of the follow-up analysis was the relationship between the patients’ mood as measured by the MDMQ and the rate of late complications (incisional hernia, wound healing disorders, and functional disorders).

The statistical analysis was performed using IBM SPSS (version 29.0). All the results were tested for normal distribution, resulting in a Student’s *t*-test or Wilcoxon rank sum test for the comparison of groups (patients with vs. patients without complications). A paired *t*-test was used for paired samples, as all the results showed a normal distribution. Categorical data were evaluated by a Chi-squared test. The numerical data are presented as mean and standard deviation. The categorical data are given as absolute numbers and as percentage of all the data. Repeated measures ANOVA (taking into account preoperative, 3rd day, and follow-up results due to the completeness of data) was used to evaluate the overall effects. The covariates taken into account for repeated measures ANOVA were sex, age, and the diagnosis of cancer. The significance level was set to two-sided α = 0.05. The results have been corrected according to the Holm–Bonferroni method due to multiple testing.

## 3. Results

**Figure 2:**
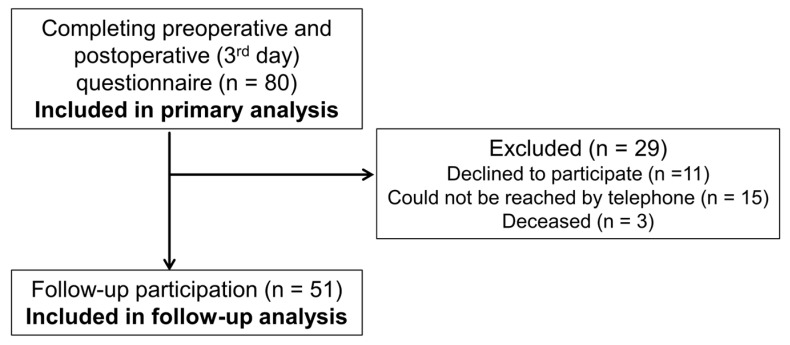
Flow of participating patients.

Of the original 80 patients, 51 patients participated in the follow-up study (Figure 2). Three patients had died in the meantime, which made it impossible to obtain information about their physical or emotional situation after surgery. No data could be collected from 26 patients as they either did not wish to participate (n = 11) or could not be reached by telephone several times (n = 15). The follow-up took place on average 30.3 ± 5.3 (17–38) months after the operation.

Of the 51 patients reached, 45% (n = 23) were male and 55% (n = 28) were female. The patients were on average 59 years (18–84 years) old. Compared to the initial study, the follow-up patients were on average the same age, but slightly more often female (49% vs. 55%) [27]. All the characteristics of the follow-up patients are shown in Table 1.

Satisfaction with the result of the operation was rated with an average of 8.5 ± 2.3 (1–10) points. The current state of health was assessed with an average of 6.8 ± 2.3 (3–10) points. The majority of the follow-up patients (80%, n = 41) did not feel that their quality of life had been impaired by the previous operation. The patients who stated that their quality of life had been impaired since the operation reported significantly lower satisfaction with the result of the operation (6.7 ± 3.2 vs. 8.9 ± 1.8 points, *p* = 0.010). There was no significant difference in the health status of the patients who stated that their quality of life had been impaired since the operation (5.8 ± 2.4 vs. 7.0 ± 2.3 points, *p* = 0.103).

### 3.1. Results of the Multidimensional Questionnaire (MDMQ)

**Figure 3:**
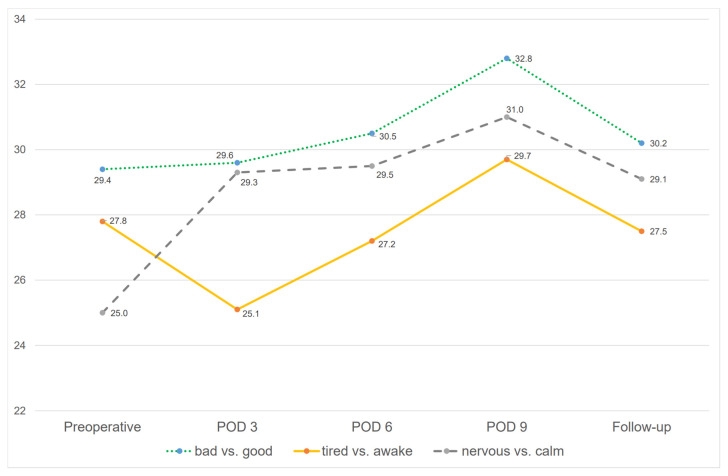
Results of the multidimensional questionnaire (MDMQ) from preoperative to follow-up, green dotted line: mood (bad vs. good); orange line: wakefulness (tired vs. awake); grey striped line: arousal (nervous vs. calm); POD = postoperative day. The lower the value, the more the feeling is shifted towards bad, tired, and nervous. Repeated measures ANOVA (considering preoperative, POD3, and follow-up results due to the completeness of data) revealed a statistically significant effect of time on patients’ wakefulness (*F*(2.0,94.0) = 3.8, *p* = 0.026).

The course of the MDMQ is shown in Figure 3 and in Table 2.

Preoperatively, the average scores of mood (bad vs. good), wakefulness (awake vs. tired), and arousal (nervous vs. calm) were 29.4 ± 7.0 (4–39) points, 27.8 ± 7.3 (12–40) points, and 25.0 ± 6.2 (11–36) points, respectively. The follow-up revealed an average mood score of 30.2 ± 6.9 (13–40) points, an average wakefulness score of 27.5 ± 7.9 (8–39) points, and an average arousal score of 29.1 ± 6.5 (8–39) points. The level of mood showed a significant increase between the 6th and 9th postoperative days (30.5 vs. 32.8, *p* = 0.005). The level of wakefulness differed significantly between the preoperative score and the postoperative day 3 score (27.8 vs. 25.1, *p* = 0.045) as well as between day 3 and day 6 (25.1 vs. 27.2, *p* = 0.027) and between day 6 and day 9 (29.5 vs. 31.0, *p* = 0.010). The level of arousal differed significantly between the preoperative score and the postoperative day scores (3rd day: 25.0 vs. 29.3, *p* < 0.001; 6th day: 25.0 vs. 29.5, *p* < 0.001; 9th day: 25.0 vs. 31.0, *p* = 0.036) as well as between the preoperative score and the follow-up score (25.0 vs. 29.1 points, *p* < 0.001). Repeated measures ANOVA considering sex, age, and diagnosis of cancer as covariates did not reveal statistically significant differences in time for mood (*F*(2.0,94.0) = 2.3, *p* = 0.111) and arousal (*F*(1.7,81.4) = 2.7, *p* = 0.080). There was a statistically significant effect of time on patients’ wakefulness (*F*(2.0,94.0) = 3.8, *p* = 0.026).

### 3.2. Late Complications and Associations with MDMQ Results, Health Status and Satisfaction Score

At follow-up, a total of 37 patients (73%) rated their further postoperative course after discharge as completely free of complications. Fourteen patients (28%) stated a late complication after discharge. Seven patients (14%) reported the development of an incisional hernia. Two patients (4%) stated a wound-healing disorder. One patient (2%) suffered from a late anastomotic leakage. Four patients (10%) reported other complaints such as changes in bowel habits or recurrent abdominal pain. As previously defined, we only categorized postoperative changes that were stressful for the patients or that led to follow-up interventions as late complications. Overall, a total of 20 patients reported changes in their bowel habits, but only 3 patients considered this to be a stressful consequence of the surgery. Three patients (6%) stated that they had developed new food intolerances since the operation, but none of the patients rated this intolerance as a stressful consequence of the operation. Fifteen patients (29%) reported the need for a change in diet, although none of them considered this to be a stressful consequence of the operation.

### 3.3. Occurrence of Complications and Associations with MDMQ Results and Other Potentially Influencing Factors

The dimensions of the MDMQ score did not differ significantly between the patients with and without late complications (Table 3). Repeated measures ANOVA considering sex, age, and the diagnosis of cancer as covariates and the occurrence of late complications as between-subject factors did not reveal statistically significant differences in time for mood (*F*(2.0,92.0) = 2.4, *p* = 0.096) and arousal (*F*(1.8,80.3) = 2.9, *p* = 0.068), but we found a weak significant association between the mood and the occurrence of late complications (*p* = 0.048). There was a statistically significant effect of time on patients’ wakefulness (*F*(2.0,92.0) = 3.9, *p* = 0.024).

Taking into account all the complications that occurred (both early as already reported in [32] and late complications), there was no significant difference in the results of all three MDMQ dimensions at all the measured time points between the patients with complications (n = 33) and patients without complications (n = 18). Repeated measures ANOVA considering sex, age, and the diagnosis of cancer as covariates and the occurrence of complications as between-subject factors did not reveal statistically significant differences in time for mood (*F*(2.0,92.0) = 2.4, *p* = 0.094) and arousal (*F*(1.7,79.2) = 2.7, *p* = 0.083), but of wakefulness (*F*(2.0,92.0) = 4.0, *p* = 0.022). We found no association between the occurrence of complications and the results of the MDMQ.

The patients with and without late complications did not differ significantly regarding their health status (6.14 ± 2.7 vs. 7.0 ± 2.1, *p* = 0.326) and their satisfaction score (7.6 ± 2.8 vs. 8.8 ± 2.1, *p* = 0.099). The quality of life was not significantly impaired regardless of whether a late complication had occurred or not (35% vs. 14%, *p* = 0.086). In the group of patients with late complications, no increased rates of bowel habit changes (36% vs. 27%, *p* = 0.099), changes in dietary habits (0% vs. 8%, *p* = 0.373), or new food intolerances (0% vs. 8%, *p* = 0.373) were reported.

## 4. Discussion

Diseases requiring surgical intervention are often accompanied by a high level of emotional distress, which is suggested to have negative effects on the psychological and physical outcomes after surgery. The hypothesis of the exploratory follow-up study was that perioperative emotional strain has an influence on the late postoperative complication rate. Interestingly, we found no clear association between the level of perioperative emotional distress measured by MDMQ and the occurrence of late complications, the impairment of health status, or quality of life. Although there was a weak association between mood and the presence of late postoperative complications in the repeated measured ANOVA, this result has to be evaluated very critically due to the exploratory nature of our study and the small sample size. There was a tendency towards lower MDMQ results in the patients with late complications, but these probably reflect the clinical situation of the patients rather than being a predictor of complications. In a previous study, we had already analyzed the influence of emotional distress measured by MDMQ on the short-term outcome after colorectal surgery and found no differences between patients with and without complications [32]. Together with the results of the previous study, we assume that emotional burden has no major influence on either the short or long-term outcome of our colorectal surgery patients. Nevertheless, the results are interesting as the MDMQ scores illustrate the emotional course of colorectal surgery patients.

Our study is one of the first studies using MDMQ to evaluate the emotional strain of surgical patients. The MDMQ is a commonly used questionnaire in German-speaking countries, being a good alternative for measuring emotional trends in cross-sectional studies [35]. The decision to use the MDMQ was made according to the recommendation of colleagues from the Department of Psychosomatic Medicine and Psychotherapy of our clinic due to the ease of implementation. We assumed that it would be too burdensome for patients to fill out a detailed questionnaire several times in the early postoperative period. Other questionnaires such as the SF-36, which is also very frequently used for surgical patients, appear to have difficulties in reflecting the situation of surgical patients [40]. Moreover, the SF-36 is not intended to measure a patient’s mood, it is rather an overview of the overall situation, which was not the aim of our study. In our study, the MDMQ demonstrated its applicability in surgical patients, as a large proportion of patients were able to use the questionnaire over several time points. At the follow-up, the mood results of the surgical patients have returned to those of the normal population (30 vs. 30 points), but there are still slight differences in wakefulness (28 vs. 26 points) and arousal (29 vs. 28 points) [35]. Unsurprisingly, the MDMQ emphasizes the change in patient arousal over time. Preoperatively, many patients are tense, which corresponds to the emotional strain of the potentially life-threatening situation; in the postoperative course, there is then a relaxation with above-average values, which gradually return to normal during the follow-up examination. The change in patients’ wakefulness is also unsurprising, as more tiredness was reported early after the operation than at later times. Thus, the plausibility of the results supports the applicability of the MDMQ in surgical patients. Nevertheless, the MDMQ must prove its validity for surgical patients in future projects.

The results of our study could be limited by the small sample size of the exploratory follow-up. Although the planned sample size was reached for the initial study, the follow-up was a smaller sample size analysis due to feasibility reasons. Of the initial 80 patients, it was only possible to reach 62 patients (78%) by telephone. Of the patients reached, 11 were not willing to take part in the study. This is a disadvantage, as it can be assumed that these patients were potentially not interested in further contact with us due to negative experiences in the postoperative course, perhaps also due to complications. Tolonen et al. pointed out that older patients sometimes feel too ill to take part in a study [41]. Thus, it could be possible that the real complication rate of our patients was higher than what we have now been able to report. As the patients’ consent to the follow-up was necessary for ethical reasons, it was not possible for us to reconstruct the complication data from other sources (e.g., via the patient’s family doctor). This problem affects our study, as it is assumable that patients with negative experiences tend to withdraw their consent to research [42,43]. On the other hand, contact with our study staff is also a chance to say that things have gone badly.

A multicenter study by the CHIR-Net SIGMA Study Group examined the course of 347 patients for up to 6 months after major cancer surgery using PRO-CTCAE^®^ [44]. While most symptoms were only weakly correlated with perioperative complications, specific problems such as the loss of appetite and constipation were significantly associated with serious complications at early postoperative time points. In addition, symptoms such as loss of appetite, nausea, and concentration problems were significantly associated with an increased risk of death within six months. It is interesting that even small, seemingly negligible complaints such as the loss of appetite were associated with an increased risk of death. Dumitra et al. showed that the patients’ quality of life after elective colorectal surgery was affected by the occurrence of complications [45]. Similarly, our study showed a tendency for lower mood scores in our patients with complications. It is plausible that patients with complications have a bad mood and a lower quality of life after surgery. Furthermore, it is plausible that patients’ mood is influenced by past experiences and pre-existing conditions. A study by Svensson et al. emphasizes the role of hope for the regaining of health in preoperative anxiety of patients to stabilize mood and anxiety before surgery [46]. About half of the patients had undergone surgery before the study, which is why positive or negative past experiences could have influenced the results of the questionnaire. When patients lose hope and surgery is a necessary evil, it is possible that their mood will deteriorate. In future studies, it should also be of interest to ask about the reasons for mood impairment and to understand more precisely why surgical patients feel what they feel.

As already mentioned in the introduction of this manuscript, recent research indicates that preoperative anxiety and depression might have an influence on long-term outcomes after surgery [21,24]. Questions arise as to why we were unable to make this observation in our study. In addition to the methodological factors already mentioned above, our study shows that patients’ preoperative mood differed only a little from the general population [35]. In a study by Perski et al. with cardiac surgery patients, the emotional well-being of patients before surgery was significantly reduced compared to the general population [47]. In another study by McHugh et al., who examined the outcome of patients after hip replacement surgery, the anxiety and depression scores before surgery also tended to be higher than in the general population [48]. Unlike other surgical studies, which often use specific questionnaires for anxiety and depression, the MDMQ is a mood questionnaire that does not specifically address anxiety and depression. Since our study is the first to apply the MDMQ to surgical patients, it is, therefore, possible that the MDMQ is not able to capture the relevant emotional dimension for postoperative recovery.

It is interesting that the grade of satisfaction after the operation was rated 8.5 out of 10, and that 80% of the patients reported no reduction in their quality of life after the operation. Leonhardt et al. reported the satisfaction with medical treatment of non-migrant and migrant colorectal cancer patients, and found scores between 7 and 8 points on a Likert scale, which is lower than the results of our patients [49]. Overall, it is difficult to compare the results of a Likert scale with other publications as most of the recent studies measure patient satisfaction usually with complex survey instruments. Nevertheless, our simple questions provide an insight into our patients’ feelings about their previous surgery, but if satisfaction and quality of life had been our primary objectives, we would have preferred a standardized questionnaire in order to obtain more information about our patients’ perceptions. Patient satisfaction and patient-reported outcome measures (PROMs) have recently become an increasingly important research topic in surgery [50]. PROMs are validated questionnaires to assess quality of life, functionality, and symptoms from the patient’s perspective. To put it bluntly, the question is no longer just whether you survive a surgical procedure, but also how you survive it. Although we were unable to show a clear correlation between the preoperative emotional strain of the patients and the postoperative outcome in our study, emotional stress should not be neglected in the future either. Preoperative emotional distress may not be predictive of later complications after colorectal surgery, but complications do have a significant impact on emotional well-being, quality of life, and functional outcome after surgery. Fortunately, a large proportion of our study patients seem to have survived the operation well and do not experience any stressful restrictions in their future lives. It is important to note that there are also patients who do not feel the same way. Our study illustrates that there are patients who were unwell before the operation, during the postoperative course, and also during the follow-up. It is our medical duty to pay attention to these patients, to be aware of them, and to offer them our help.

## 5. Conclusions

The results of the standardized multidimensional mood questionnaire (MDMQ) for measuring the perioperative emotional strain of patients undergoing colorectal surgery do not appear to have any correlation with the development of late postoperative complications. Nevertheless, the results are interesting and indicate the sometimes high resilience and vulnerability of individual patients. Future research should not only consider the physical well-being before and after colorectal surgery, but also the emotional health of patients in order to be able to take this into account during the recovery process. Furthermore, the role of pre-existing psychiatric illness, socioeconomic status, and social support during emotional and physical recovery from surgery should be further investigated.

## Figures and Tables

**Table 1:** Baseline characteristics of all the follow-up patients (n = 51), obtained at the time of study inclusion (i.e., before surgery).

	Median ± SD (Range)
**Age (years)**	58.8 ± 17.4 (18–84)
**BMI (kg/m^2^)**	24.6 ± 5.4 (18–40)
	**n (%)**
**Gender (male/female)**	23 (45%)/28 (55%)
**Type of surgery**	
Left hemicolectomy	1 (2%)
Right hemicolectomy	16 (31%)
Removal of the sigmoid colon	22 (43%)
Gastrointestinal continuity restoration	5 (10%)
Ileocoecal resection	3 (6%)
Recreation of illeotransversostomy	2 (4%)
Subtotal colectomy	1 (2%)
Segmental bowel resection	1 (2%)
**Surgery for the first time**	25 (49%)
**Surgical access**	
Laparoscopic	34 (67%)
Laparotomy	17 (33%)
**Pre-existing illness**	
Chronic inflammatory bowel disease	7 (14%)
Current cancer	19 (37%)
Previous cancer	8 (16%)
Cardiovascular disease	23 (45%)
Diabetes	3 (6%)
Renal insufficiency	2 (4%)
**Smoker**	
Yes	8 (16%)
No	25 (49%)
Quitted	18 (35%)
**Alcohol**	
Regularly	6 (12%)
Occasionally	33 (65%)
No	12 (23%)
Drug consumption *	1 (2%)
**Diet**	
Omnivore	49 (96%)
Vegetarian	1 (2%)
Other	1 (2%)

(SD = standard deviation; * one patient stated regular consumption of cannabis).

**Table 2:** Results of the multidimensional questionnaire (MDMQ) before the operation; on the 3rd, 6th, and 9th postoperative day; and during follow-up.

MDMQ Scores (±SD, Range)	Preoperative (n = 51)	3rd Day (n = 51)	6th Day (n = 37 ^+^)	9th Day (n = 10 ^+^)	Follow-Up (n = 51)	*p* *
Mood (bad vs. good)	29.4 (±7.0, 04–39)	29.6 (±6.2, 19–40)	30.5 (±5.8, 18–40)	32.8 (±5.2, 21–38)	30.2 (±6.9, 13–40)	0.539
Wakefulness (awake vs. tired)	27.8 (±7.2, 12–40)	25.1 (±7.2, 12–39)	27.2 (±6.2, 17–39)	29.7 (±7.3, 16–36)	27.5 (±7.9, 08–39)	0.809
Arousal (nervous vs. calm)	25.0 (±6.2, 11–36)	29.3 (±6.1, 12–39)	29.5 (±5.5, 17–40)	31.0 (±5.2, 23–38)	29.1 (±6.5, 08–39)	**<0.001**

SD = standard deviation, ^+^ missing patients were already discharged. * paired *t*-test to compare the preoperative score and the follow-up score.

**Table 3:** Comparison of the MDMQ results of the patients with (n = 14) and without (n = 37) late complications during the observational period.

MDMQ Scores (±SD, Range)	Patients with Late Complications	Patients Without Late Complications	*p* *
**Mood (bad vs. good)**			
- preoperative	26.1 ± 9.1 (04–38)	30.6 ± 5.8 (10–39)	0.107
- 3rd day	27.5 ± 5.9 (19–39)	30.4 ± 6.2 (19–40)	0.145
- 6th day ^+^	29.1 ± 5.0 (18–35)	31.0 ± 6.1 (19–40)	0.356
- 9th day ^+^	32.7 ± 5.0 (29–38)	32.9 ± 5.6 (21–37)	0.384
- follow-up	29.4 ± 7.1 (19–40)	30.5 ± 6.9 (13–40)	0.639
**Wakefulness (awake vs. tired)**			
- preoperative	26.0 ± 5.9 (15–36)	28.5 ± 7.7 (12–40)	0.280
- 3rd day	22.6 ± 7.7 (13–35)	26.1 ± 6.9 (12–39)	0.121
- 6th day ^+^	27.0 ± 4.7 (20–33)	27.3 ± 6.8 (17–39)	0.892
- 9th day ^+^	33.0 ± 5.2 (29–36)	28.3 ± 8.0 (16–36)	0.384
- follow-up	26.6 ± 9.1 (09–39)	27.9 ± 7.5 (09–39)	0.607
**Arousal (nervous vs. calm)**			
- preoperative	23.6 ± 6.5 (14–34)	25.5 ± 6.1 (11–36)	0.335
- 3rd day	26.9 ± 7.9 (12–39)	30.1 ± 5.1 (18–39)	0.150
- 6th day ^+^	28.1 ± 4.6 (17–35)	30.2 ± 5.8 (20–40)	0.300
- 9th day ^+^	30.0 ± 6.2 (25–37)	31.4 ± 5.2 (23–38)	0.716
- follow-up	29.1 ± 6.2 (21–39)	29.0 ± 6.7 (16–40)	0.956

SD = standard deviation, * *t*-test. ^+^ n is reduced to 11/26 (with/without late complication) on day 6 and to 3/7 on day 9 due to patients’ discharge.

## Data Availability

The data presented in this study are available upon request from the corresponding author due to ethical restrictions.
